# Tuberculous meningitis: a 10-year case analysis of critical care admissions

**DOI:** 10.1186/cc13638

**Published:** 2014-03-17

**Authors:** N Ojukwu, C Talati, S Wijayatilake, G De la Cerda, A Bellini, R Jain

**Affiliations:** 1 Queen's Hospital, London, UK

## Introduction

Tuberculous meningitis (TBM) is the least common extrapulmonary manifestation of tuberculosis. Although the UK incidence of TBM is relatively low, it carries a high mortality and morbidity [1]. Neurological deterioration continues to be an important reason for ICU admission [2]. Little is known about the outcomes for TBM patients requiring ICU admission. Our aim is to evaluate patient demographics, tBm clinical data, and the necessity for organ support, and whether this can be associated with outcome.

## Methods

A retrospective study at a tertiary centre of patients with TBM admitted to our ICU between 2000 and 2012. Data were retrieved on demographics, microbiology, radiology and pharmacological findings and type and level of organ support. APACHE II and SOFA scores were calculated. Patients were stratified into two groups: CSF PCR+ve and CSF PCR-ve.

## Results

Eight patients (six males:two females) were evaluated. Total mean age was 43.9 ± 8.09 years. A reduction in GCS was the main reason for ICU admission. All patients received ≥3 anti-TBM drugs and steroids. Two CSF PCR+ve patients had primary drug resistance to isoniazid. There was also a longer mean delay in the time of onset of anti-TBM treatment in CSF PCR+ve patients (4 ± 2.88 days). Higher APACHE II and SOFA scores on admission (mean = 23, 8.5) were associated with a positive CSF PCR result. Increased requirements for mechanical ventilation (100%), tracheostomy (50%), inotropes (75%), neurosurgical intervention (predominantly CSF drainage) (100%) and enteral feeding (50%) were all significant for this group. Mortality and long-term neurological morbidity were substantially higher for PCR+ve patients (75% and 25%). In contrast, the majority of culture-negative patients survived (75%), and experienced good recoveries at follow-up. Overall mortality was 50%.

## Conclusion

This is the second documented case analysis of TBM and ICU admission in adult patients [1,2]. Findings show that poor outcomes are associated with positive CSF PCR results. Factors linked to poor outcomes include delays in initiating anti-TBM treatment, neurosurgical interventions, and an increased requirement for multiorgan support. Earlier drug susceptibility testing would be preferable particularly for patients with positive CSF PCR cultures. Given the potential severity of TBM, a high index of clinical suspicion remains critical towards optimizing outcomes.

## References

[B1] Pehlivanoglu F Scientific World Journal 2012 2012 16902822619611

[B2] Verdon R Clin Infect Dis 1996 22 982 988 10.1093/clinids/22.6.982 8783697

